# Supplementary material for: Splice-Junction-Based Mapping of Alternative Isoforms in the Human Proteome
Source: Cell Rep. Author manuscript; Available in PMC 2020 Jan 15. (PMC6961840; doi:10.1016/j.celrep.2019.11.026)
Supplement: 2 [file NIHMS1546469-supplement-2.zip › DF1/PXD010154/Esophagus/Esophagus_25_RTN2_AQGGTAGVGGGPDAGPR.pdf]

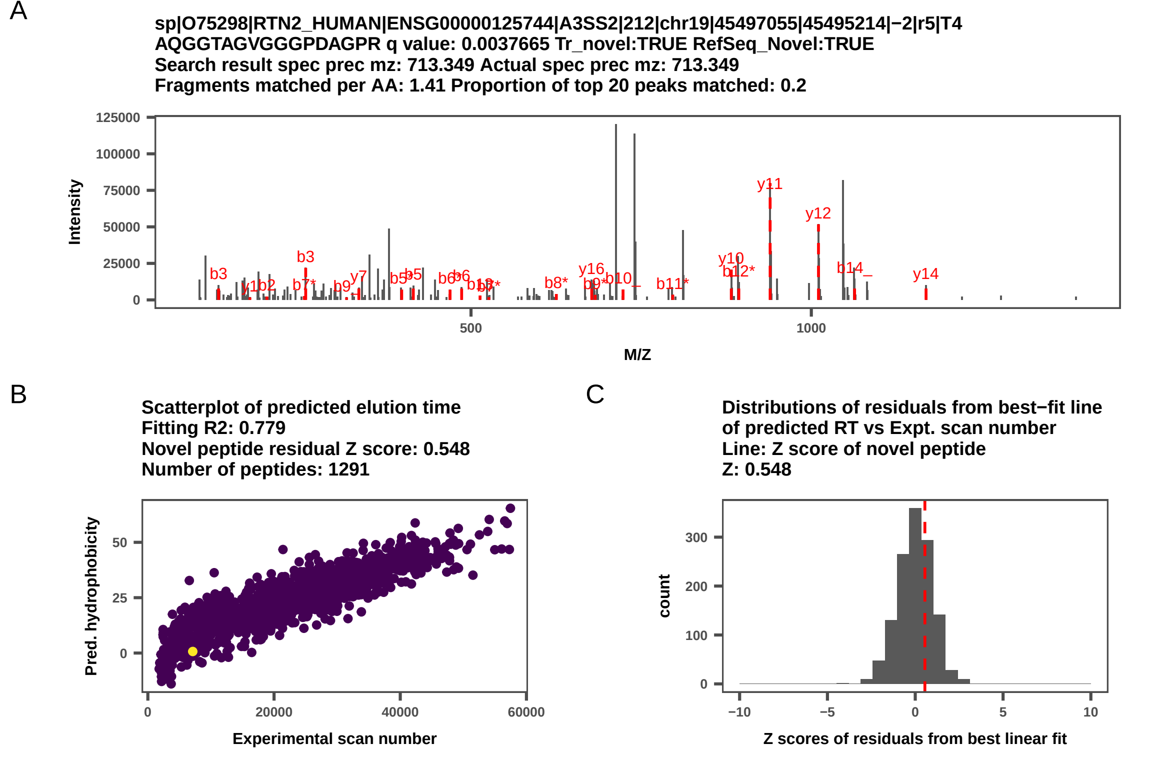

A
sp|O75298|RTN2_HUMAN|ENSG00000125744|A3SS2|212|chr19|45497055|45495214|−2|r5|T4
AQGGTAGVGGGPDAGPR q value: 0.0037665 Tr_novel:TRUE RefSeq_Novel:TRUE
Search result spec prec mz: 713.349 Actual spec prec mz: 713.349
Fragments matched per AA: 1.41 Proportion of top 20 peaks matched: 0.2
Intensity
125000
100000
75000
50000
25000
0
y11
y12
b3
y10
b12*
b14_
y14
y16
b10_
b11*
b8*
M/Z
500
1000
B
Scatterplot of predicted elution time
Fitting R2: 0.779
Novel peptide residual Z score: 0.548
Number of peptides: 1291
Pred. hydrophobicity
50
25
0
0
20000
40000
60000
Experimental scan number
C
Distributions of residuals from best−fit line
of predicted RT vs Expt. scan number
Line: Z score of novel peptide
Z: 0.548
count
300
200
100
0
−10
−5
0
5
10
Z scores of residuals from best linear fit
